# A Perceptual Approach to the Impact of CSR on Organizational Financial Performance

**DOI:** 10.3390/bs13050359

**Published:** 2023-04-25

**Authors:** Marian Cazacu, Simona Dumitriu, Iulian Georgescu, Dorel Berceanu, Dalia Simion, Anca Antoaneta Vărzaru, Claudiu George Bocean

**Affiliations:** 1 Doctoral School, University of Craiova, 13 AI Cuza Street, 200585 Craiova, Romania; cazacumariantbm@gmail.com (M.C.); simonadumitriu1969@gmail.com (S.D.); 2 Department of Finance, Banking, and Economic Analysis, Faculty of Economics and Business Administration, University of Craiova, 13 AI Cuza Street, 200585 Craiova, Romania; dorel.berceanu@edu.ucv.ro (D.B.); dalia.simion@edu.ucv.ro (D.S.); 3 Department of Economics, Accounting and International Business, Faculty of Economics and Business Administration, University of Craiova, 13 AI Cuza Street, 200585 Craiova, Romania; anca.varzaru@edu.ucv.ro; 4 Department of Management, Marketing and Business Administration, Faculty of Economics and Business Administration, University of Craiova, 13 AI Cuza Street, 200585 Craiova, Romania

**Keywords:** social responsibility, CSR, financial performance, reputation, employees’ attraction and retention, clients’ attraction and loyalty, access to capital

## Abstract

Corporate social responsibility (CSR) is a progressively significant issue for organizations and governments. To benefit from a good reputation that reflects on organizational performance, organizations must ensure the balance between stakeholders’ needs. This paper studies the direct and indirect effects of CSR on organizational financial performance as perceived by employees of organizations. The investigation used structural equation modeling to evaluate and describe the nature of the relationship between these two variables. The empirical study uses a perceptual approach, evaluating the perceptions of the closest stakeholders (employees). Data on the perceptions of 431 employees in Romanian organizations were collected following a questionnaire-based survey. The results indicate a strong effect of social responsibility on both direct and mediated organizational financial performance. The relationships established with the stakeholders ultimately affect organizational financial performance through variables such as the attraction and retention of employees, the attraction and loyalty of customers, more accessible access to capital, and the organization’s reputation.

## 1. Introduction

In recent decades, organizations began implementing strategic management practices concerning social responsibility to achieve the objective of organizational sustainability [1,2]. Social responsibility reveals the commitment of organizations to consider not only economic drivers but also social and environmental drivers [1,3]. This practice has increasingly expanded, becoming one of the pillars of organizational sustainability [4,5].

Organizations’ financial results were, until the emergence of stakeholder theory, the determining indicator for organizational value and its long-term success. Maximizing shareholder wealth has long been the central objective of private-sector organizations. Since the 1990s, the organizational paradigm has translated from maximizing profits to the balance between stakeholders’ needs. If, in the beginning, CSR was just a marketing image exercise, nowadays the public image of CSR has changed dramatically, becoming a strategic practice [6]. Organizational performance is no longer valued only by financial measures but is also measured by nonfinancial actions that reveal organizations’ contributions to society [7]. CSR targets all categories of stakeholders, not just shareholders, and customers, who were organizations’ focus in the past [8,9,10].

To achieve the objectives of sustainability, organizations must behave responsibly, as this will bring them additional intangible benefits (improving the image of theorganization) and an increase in financial performance [11,12], which are vectors of sustainable development because they influence the level of perception [13]. Therefore, organizational reputation management involves the continuous management of a balance between internal perceptions and external expectations [14,15]. Nowadays, an organization is no longer identified only by its object of activity but instead is identified by how it offers products and services, which leads to a reputation that influences financial results [16]. Thus, the perceptions of different categories of stakeholders regarding the organization’s responsible and ethical way of acting influence its reputation among employees, customers, investors, and the community, leading to the organization’s financial sustainability [17].

CSR actions lead to a better image and an increased reputation in the perceptions of clients, employees, investors, and the community [18,19]. Customer loyalty can be ensured, first, by the quality of products and services and, second, by an improved reputation along with a positive image that attracts new customers and keeps old ones [20]. Since the reputation of managers is related to the importance and image of organizations [5], they will support the implementation of social responsibility programs and ethical business conduct [21].

The improvement of organizational financial performance is the primary concern of social responsibility initiatives [22,23], so it is necessary to demonstrate the influence of CSR on financial performance, both directly and mediated through intangible variables related to the various categories of stakeholders (attraction and retention of employees, customer attraction and loyalty, more accessible access to capital, increased reputation). Empirical research based on accounting, financial, and market indicators cannot estimate the effects of intangibles on financial performance except through second-round financial and accounting indicators (the effects are uncertain because financial and accounting indicators are synthetic and encompass multiple effects). To address this gap, the empirical study uses a perceptual approach, evaluating the perceptions of the closest stakeholders (employees), who know best the organization’s CSR actions and generic financial indicators (turnover and profit). The advantage of evaluating employees’ perceptions also appears from their relationships with all the organization’s stakeholders [24,25]. Implementing CSR actions with a high level and high regularity can generate better financial performance directly and through moderating variables. The theoretical base applied in the paper is primarily rooted in stakeholder theory [24]. The study also draws on the concept of perceived corporate social responsibility, which suggests that stakeholders’ perceptions of a company’s CSR initiatives are essential in shaping their attitudes and behaviors towards the company [14]. Another theoretical approach is the resource-based view of the firm, which argues that organizations possess unique resources and capabilities that provide a competitive advantage [22]. In this view, socially responsible behavior perceived by employees is a valuable resource that can differentiate an organization from its competitors and lead to superior financial performance [21,22,25].

Based on a perceptual approach to these concepts, this paper offers a model for evaluating the direct and mediated relationships between social responsibility and organizational financial performance. The work has a structure composed of six sections. The first two sections present the research topic and other researchers’ views. The following two sections present the research methodology and the results obtained. Finally, the last two sections discuss the results and conclusions.

## 2. Literature Review and Hypothesis Development

As a result of a turbulent, highly competitive global environment, organizations face multiple challenges regarding operational sustainability [26]. Organizations must not have profit maximization as their sole objective for having a sustainable path but must also consider environmental protection and social issues arising from organizational operations [27]. Social responsibility has been debated since the last decades of the 20th century, becoming highly publicized [28]. Social responsibility has also come to the attention of various international bodies that have proposed standards and guidelines in the field. In addition to the global perspective, many countries have developed relevant norms to promote the implementation of social responsibility at the corporate level [29]. These normative documents emphasize the voluntary nature of social responsibility programs but encourage such programs because organizations can contribute to solving many environmental and social problems [30].

Furthermore, a vision based on social responsibility requires organizations to have a more excellent orientation towards all stakeholders, giving importance to investors and other categories of stakeholders. The crisis generated by the COVID-19 pandemic strengthened this orientation, emphasizing the role of organizations in solving problems not only of an economic nature but also those involving social and environmental issues [31]. An organization’s social responsibility program improves its reputation and financial position and provides additional advantages for its sustainable development.

Social responsibility has been at the midpoint of public attention, but questions are raised about the tangible benefits it brings to the organization, especially regarding organizational financial performance. The relationship between responsibility and financial performance has been studied for a long time, through various approaches, in the economic literature, with three different points of view [29,32,33,34,35,36,37,38,39,40,41]. The first approach is the neoclassical one, in which excessive spending on implementing social responsibility programs encumbers corporate profits, significantly reducing them [32]. In addition, amplifying conflicts between stakeholders by prioritizing some at the expense of others will increase costs, reducing financial profitability [33]. A second approach assumes that greater social responsibility of organizations will lead to increased organizational reputation, attracting and retaining the best talent, attracting and retaining customers, and improving access to capital due to the organization’s ethical conduct. [29,34,35]. This vision requires a robust communication activity of the social responsibility actions and the disclosure of social responsibility reports and sustainability reports that reveal all activities and results following international guidelines and standards. Therefore, many large companies invest significant resources to actively assume social responsibility and disclose their CSR activities [36,37]. Finally, a third approach considers that causal relationships cannot be drawn between activities in social responsibility programs and organizational financial performance [38].

Various studies have identified positive, negative, nonlinear, and no correlations between social responsibility and organizational financial performance. Many of these results are due to using different indicators to measure the two concepts [29]. However, most of the researchers believe that CSR can lead to the improvement of financial performance, either directly but especially by mediating some variables related to the organization’s stakeholders, such as attracting and retaining employees, client loyalty, more accessible access to capital, and the increased reputation of the organization [42,43]. Since the last century, Cochran and Wood [44] have demonstrated that an organization with a production process that implements the CSR principles will have improved financial performance compared to organizations guided by the principles of profit maximization. Stakeholder theory has shed light on the mechanisms through which social responsibility programs can contribute to improving financial performance. The attention given to stakeholders’ needs makes them favorable in the relationship with the organization and in the relationships between the groups of stakeholders, which reduces organizational tensions. [45]. Mitigating conflicts between stakeholders and their satisfaction determines improving reputation, minimizing costs, and obtaining competitive advantages that positively influence financial performance [46,47]. However, researchers and followers of the agency theory believe that excessive investments in CSR do not bring marginal profit but do contribute to decreasing organizational financial performance, even if such investments bring intangible benefits related to the image and reputation of the organization [48,49]. There is also a category of empirical research that has demonstrated with the use of various sets of variables and different methods that CSR and financial performance do not correlate [50] and that there is a nonlinear correlation in which many external variables interfere with moderation and mediation effects [51].

Based on all these considerations, the first research hypothesis, H1, is the following:

**Hypothesis** **H1.** *CSR exerts a medium direct positive influence on organizational financial performance*.

Most researchers do not consider the influence of other variables on financial performance, influenced by the level and regularity of CSR actions [16]. However, the mediating effects can be significant, which affects the research results of CSR influences on organizational financial performance because CSR also has intangible benefits that contribute to tangible results (increased financial performance). Therefore, many researchers consider the mediating effects a field that deserves to be expanded in the future [5,52,53]. Employee perception is significant because it influences organization’s reputation, motivating the organization to invest in responsible actions with a positive impact on all stakeholders [54]. The level and regularity of CSR actions must be permanently communicated to all categories of stakeholders to increase the organization’s reputation among them [52]. Through transparency and speed of communications, organizations ensure an excellent image and obtain, as a consequence, legitimacy and reputation, benefiting from a competitive advantage in goods, services, labor, capital markets, etc. [55]. Reputation contributes to employees’ attraction and retention, clients’ attraction and loyalty, and more accessible access to capital, increasing the financial sustainability of the organization [56]. However, reputation is an intangible asset that generates a competitive advantage through the organization’s image in the relationship with its stakeholders [57]. In recent years, there have been increasing cases of organizations whose reputations have suffered due to the impact of social networks among the public [5].

Based on these considerations, the hypothesis of the H2 research is the following:

**Hypothesis** **H2.** *CSR exerts a substantial direct positive influence on employee attraction and retention, client attraction and loyalty, more accessible access to capital, and reputation*.

Some opinions show that despite the additional expenses, CSR generates additional income through improved image and reputation that exceeds the total amount spent on CSR programs [58]. On the other hand, economists of neoclassical origin see CSR only as a burden for the organization, with the amounts spent on CSR not generating marginal profit [59]. There is no consensus in the literature, which means that the relationship between social responsibility and financial performance is still under discussion [60,61]. Different measurement frameworks based on accounting, market, and respondent perception can be encountered in various works.

Although there is a debate about the profitability of spending for social responsibility actions especially in the short term, the results of recent investigations that consider other mediating variables and the long term show positive linkages between social responsibility and financial performance [62,63].

If only the investors’ interests are considered, we can say that the relationship is primarily negative or unclear. The emergence of stakeholder theory introduced into the equation of organizational performance a series of intangible variables related to stakeholders other than investors that ultimately positively influence organizational financial performance if the organization adequately manages them. Internalizing all stakeholders links social responsibility and financial performance more noticeably [43].

The financial and managerial literature is rich, including an examination of positive relations between social responsibility and employee attraction, satisfaction and retention [64,65,66], and consumer attraction and loyalty [67,68] as well as examinations of financial performance [69], individual performance [70], and improving employee relations [71].

Employees represent the most critical category of internal stakeholders, because the organization’s added value is obtained mainly with the help of their work and creativity and the spirit of innovation that characterizes these stakeholders [23,72]. Therefore, organizations must pay special attention to employees. Otherwise, their involvement and commitment will decrease significantly [73]. Employees can influence the productivity of an organization, their involvement being essential to financial performance [74]. Various researchers indicate that social responsibility positively impacts employee performance, increasing employee attraction and retention, including through actions supporting environmental conservation [75]. The involvement of employees in social responsibility actions and transparent communications increases the organization’s reputation among employees. The financial performance of an organization also depends on the support of the community from which the employees come [23]. A high-level regular CSR program helps an organization create a better reputation among the communities in which it operates [76]. An increased reputation attracts higher sales, more involved employees, and more investors, influencing the organization’s financial performance [77].

Client attraction and loyalty influence a company’s sales and, implicitly, financial performance [78]. Therefore, the organization must behave ethically and responsibly in its customer relationship. When this happens, customers also promote the business. This approach is known as consumer–organization identification, whereby customers identify themselves with the organization [79,80]. There is a direct correlation between customer loyalty and perceived service quality, which can be strengthened by the organization’s reputation for social responsibility [81]. Unethical behavior coupled with a lack of social responsibility influences consumers’ buying impulses, dramatically affecting the organization’s financial performance [82]. Customers’ interest in products and services obtained responsibly has led organizations to invest more in CSR programs [23,83]. High market competitiveness creates the need for differentiation among organizations, including in the area of social responsibility actions [84]. Moreover, customers can be a pressure factor regarding the level and regularity of implementing CSR actions [85].

Based on these considerations, the third research hypothesis, H3, is the following:

**Hypothesis** **H3.** *CSR exerts an indirect positive influence on financial performance, mediated by employee attraction and retention, client attraction and loyalty, more accessible access to capital, and increased reputation*.

From all the categories of stakeholders, employees were chosen for evaluating perceptions because they are subject to both the external and internal CSR communication process. External communication is carried out through sustainability reports, websites, and social networks [86,87]. However, internal communication makes employees essential stakeholders, because the organization can achieve more significant effects of CSR actions through their involvement [88].

## 3. Methodology

**Figure 1:**
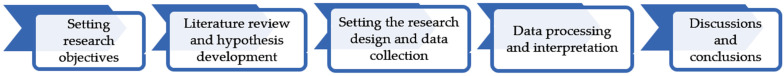
Research design. Source: developed by the authors.

The research process involved going through five stages, illustrated in Figure 1.

The empirical study involved a survey based on a questionnaire conducted between September 2022 and November 2022 among 431 employees from Romanian organizations. The study focused on large organizations (with over 250 employees) from industry and service sectors that conduct and report on their CSR activities. We used the random stratified sampling method depending on three sociodemographic variables: gender, age, and education. The respondents were assured of the confidentiality of the answers, and there was informed consent in the questionnaire. No identification data regarding the organization was requested in order to reduce biases. The sample was calculated with a confidence level of 95% and a margin of error of 4.723%. Table 1 presents frequencies for sociodemographic variables.

To measure perceptions regarding CSR, we used two items regarding the level of CSR and the regularity of CSR actions, similar to other authors in previous research [23]. Two items describe employee attraction/retention and client attraction/loyalty, as other authors have done in previous research [12,14,23]. Reputation has been assigned a single item regarding reputation level in employees’ perceptions [12,14], and access to capital involves capital lenders’ and investors’ access [14].

The validity of perceptual financial performance, as perceived by employees, has been a topic of interest in recent years. While objective financial measures such as net profit, revenue, return on assets, return on equity, and return on investment are commonly used to assess organizational financial performance, employees’ subjective financial performance evaluations are essential. Furthermore, there is an increasing recognition that using employees’ perceptions of financial performance as a metric is reliable, mainly related to HRM and CSR practices [89,90,91,92].

To measure the organizational financial performance reflected in employees’ perceptions, we used the approach of Delaney and Huselid [90] (p. 956). Therefore, we selected two recognizable indicators in organizational communications: net profit and turnover. These measures were preferred to other indicators that reflect profitability, such as ROI (return on investments), ROA (return on assets), and ROE (return on equity), because they are synthetic, explicit, and much more visible in organizational communications accessible to employees. In addition, turnover expresses total revenue, while net profit shows what is left over after deducting expenses and paying taxes and fees. For the two financial indicators, we used two items describing the perception of performance in 2022 compared to that in 2021 and the perception of performance compared to expectations. We used the five-level Likert scale, similar to other authors in previous research [12] (p. 5), [14] (p. 660), [23] (p. 7). Table 2 presents the structure, variables, and measurement scales of the questionnaire used in the empirical study.

The empirical study uses structural equation modeling (SEM) as a data processing and interpretation method. The SEM assumes the definition of unobservable latent variables based on observable variables (questionnaire items). The model used is reflexive, with the visible variables describing characteristics of the latent variables. Table 3 presents the descriptive statistics of the observable variables (questionnaire items).

**Figure 2:**
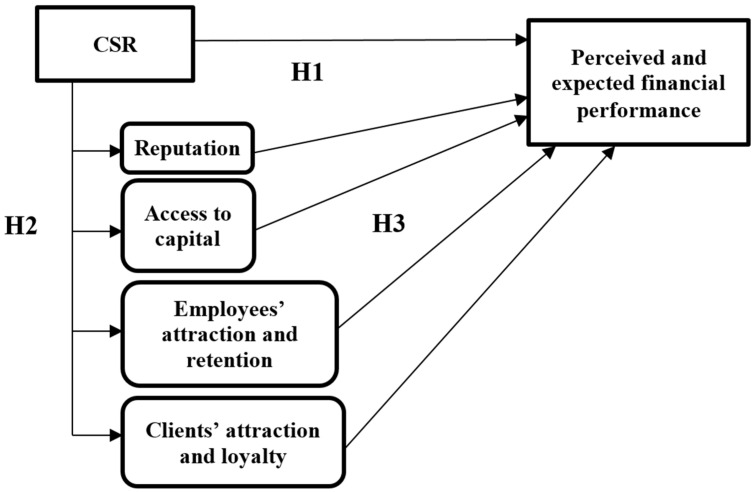
Theoretical model. Source: Developed by the authors based on literature review.

Figure 2 presents the theoretical model of the research on the direct and indirect influences of CSR on financial performance in the perception of employees. The latent variables are defined as follows: CSR, employees’ attraction and retention, clients’ attraction and loyalty, access to capital, reputation, perceived performance, and expected performance. The questionnaire items define the observable variables.

## 4. Results

**Figure 3:**
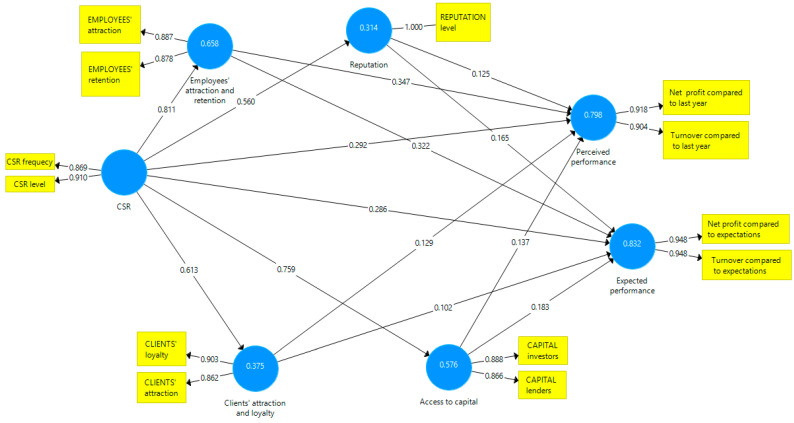
Applied model. Source: Developed by the authors using SmartPLS v.3.

We applied the PLS algorithm to the theoretical model using Smart PLS v3 software. Figure 3 displays the resulting diagram illustrating the relationships between the latent variables and the loadings of each exogenous variable. The loadings of all exogenous variables have values above 0.7, making them relevant in describing latent variables [93].

The model shows good fit (SRMR = 0.070 < 0) and measures indicating good reliability and validity of the data sets. For example, Cronbach’s Alpha has values above 0.7, Composite Reliability is above 0.8, and Average Variance Extracted is above 0.7 (Table 4).

The proposed model has good discriminant validity as well. The Fornell–Larcker criterion shows that the values on the diagonal of the discriminant matrix must be greater than all the values in the same row and column [94]. Table 5 illustrates that the model validity is excellent.

Applying a bootstrapping procedure within the model (with 500 subsamples and a significance level of 0.05) leads to obtaining the path coefficients, indicating the relationships established between the latent variables (Table 6). The relationships between the variables are significant (*p* < 0.001, t > 1.6), indicating the medium and robust effects of the independent variables on the dependent ones.

Table 6 reveals medium direct relationships of CSR on perceived performance (c = 0.292, *p* < 0.001) and expected performance (c = 0.286, *p* < 0.001), which validates Hypothesis H1. CSR exerts a medium direct positive influence on organizational financial performance. The influences of CSR on the variables regarding stakeholders are strong (c > 0.5, *p* < 0.001) (Table 6), confirming the validity of Hypothesis H2. CSR exerts a substantial direct positive influence on employee attraction and retention, client attraction and loyalty, more accessible access to capital, and the organization’s reputation.

Table 7 presents the specific indirect effects calculated after applying the bootstrapping procedure.

Table 7 reveals the indirect relationships that CSR exerts on perceived and expected performance through mediating variables, validating Hypothesis H3. The total indirect effects exerted by CSR through mediating variables on perceived performance (c = 0.534, *p* < 0.001) and expected performance (c = 0.554, *p* < 0.001) are solid. In conclusion, CSR indirectly influences organizational financial performance by mediating variables related to stakeholders. The total direct and indirect effects exerted by CSR on financial performance in employees’ perceptions are significant and robust: perceived performance (c = 0.826, *p* < 0.001) and expected performance (c = 0.840, *p* < 0.001).

The model proposed and tested with the help of PLS and bootstrapping procedures proves to have high predictive ability in evaluation of the predictive capabilities of the model. The Q^2^ value compares the prediction errors of the PLS model with the predictions of the variables’ means [93]. Table 8 shows the values obtained by the PLS prediction procedure using the SmartPLS v.3 software.

Positive values above 0.3 of the Q^2^ value indicate the high predictive capacity of the model, especially for the latent variables: perceived performance and expected performance.

## 5. Discussion

Although initially social responsibility was seen as an obligation in setting and pursuing long-term objectives, with the emergence of the sustainability concept, it also became an essential component of organizations’ sustainable development [95]. Social responsibility allows organizations to disclose nonfinancial information that is not provided by traditional accounting but is subject to the reorientation of management accounting and corporate reporting towards sustainability [29,96,97]. Sustainability accounting and reporting, especially in the digital transformation context, allow more precise quantification of social responsibility actions, offering organizations a competitive advantage [98]. As a result of the implementation of social responsibility, organizations have reoriented to increase value for stakeholders [98].

The relations between CSR and financial performance have been essential in recent decades [63,84,99,100,101,102,103]. According to research results, the benefits of CSR are numerous and mainly include improving organizations’ reputation, attracting and retaining customers, and increasing trust and access to capital. CSR significantly increases an organization’s value by increasing employee productivity, attracting and retaining the best talent, building corporate reputation, and improving stakeholder relations. Following the research results, we found that the benefits of CSR are numerous and mainly include the improvement of reputation, which is consistent with multiple studies [11,17,22,25,34], and the attraction and loyalty of customers, as other authors also state in their studies [56,67,68,72,77,80,83]. In addition, the research results showed that the benefits of CSR are related to increase in trust and access to other stakeholders, such as investors and creditors, facilitating more straightforward access to capital [104]. Therefore, CSR has a significant role in increasing organizations’ value by increasing employee productivity, attracting and retaining the best talent, attracting and retaining customers, improving access to capital, building corporate reputation, and improving relations with all stakeholders [105].

Regarding the direct and significant influence of social responsibility on organizational financial performance, the research of Hypothesis H1 demonstrated through the obtained results that in the perception of employees, social responsibility has a medium effect on perceived performance (c = 0.292, *p* < 0.001) and expected performance (c = 0.286, *p* < 0.001). This fact leads us to join other researchers regarding employee involvement in CSR actions, which can increase productivity and satisfaction and implicitly increase financial results [5,106].

By studying the influences of corporate social responsibility on employee attraction and retention (c = 0.811, *p* < 0.001), client attraction and loyalty (c = 0.613, *p* < 0.001), more accessible access to capital (c = 0.759, *p* < 0.001), and reputation (c = 0.560, *p* < 0.001), Hypothesis H2 revealed significant consistent effects in line with the findings of other empirical research [107,108]. This study also found that the level and regularity of CSR actions have a lasting impact on stakeholder perceptions, ultimately leading to improved organizational results. This aspect highlights the importance of maintaining a sustained and consistent CSR rather than a sporadic or ad hoc approach. Furthermore, this study found that CSR significantly impacted organizational reputation, which is consistent with previous research. This fact emphasizes the strategic importance of CSR in building and maintaining a positive image for organizations among various stakeholders, including employees, customers, investors, and creditors. Overall, the results of this study provide strong support for the notion that CSR can have significant positive effects on various aspects of organizational performance, including employee attraction and retention, client attraction and loyalty, access to capital, reputation, and ultimately financial results [109].

The findings from investigating Hypothesis H3 regarding mediating effect of variables related to stakeholders (employee attraction and retention, client attraction and loyalty, more accessible access to capital, and reputation) showed that employee attraction and retention exert a substantial mediating effect. In contrast, the mediating effects exerted by reputation, access to capital, customer attraction, and loyalty are relatively small but significant. Therefore, CSR exerts significant influences on these variables, which in turn strongly cumulatively influences the expected performance (c = 0.554, *p* < 0.001) and the perceived performance (c = 0.534, *p* < 0.001). Furthermore, the indirect influences exerted through the mediation effect join the direct influences, with the total effects of CSR on expected performance (c = 0.8406, *p* < 0.001) and perceived performance (c = 0.826, *p* < 0.001) being strong in the perception of employees of organizations. This study also found that the indirect influences exerted through the mediation effect join the direct influences, resulting in total solid effects of CSR on expected and perceived performance. This result suggests that organizations prioritizing CSR initiatives are more likely to achieve better performance outcomes. The study’s findings support the positive relationship between CSR and organizational performance. By investing in CSR initiatives, organizations can improve their reputation, attract and retain talented employees, and enhance customer loyalty, ultimately leading to better performance outcomes.

Research has identified social responsibility as contributing to reputation [5]. Our research findings confirmed this result, revealing CSR’s positive and significant influence on organizational reputation. Organizations must focus their CSR efforts on various stakeholders, including employees, customers, investors, and creditors, to enhance their reputation and improve their public image. [110]. CSR can positively impact an organization’s reputation, financial performance, and stakeholder relationships. Therefore, integrating CSR into business strategies and operations can be a valuable tool for organizations seeking to improve their performance and reputation. From this perspective, social responsibility can be a strategic tool that, coupled with managerial accounting and adequate sustainability reporting, leads to a good reputation and sustainable financial results [11,12,13,14,15,16,17,20,21,111,112].

### 5.1. Theoretical Implications

Management, accounting, and finance researchers consider social responsibility an essential research topic because an increasing number of organizations carry out CSR actions strategically [102,113]. In terms of financial performance, spending on CSR programs results in several direct benefits from various sources such as easy access to capital, low financing costs, attracting and retaining employees, and client loyalty [5,23,114,115]. The complexity of the CSR concept has led to a rich literature on the effects of the level and regularity of CSR actions on employee attraction, satisfaction, and retention [101,102,116]; consumer attraction and loyalty [56,67,68,72,77,80,83]; corporate image and reputation [101]; and financial performance [6,7,23]. While organizations have widely adopted CSR, there is still debate over its effectiveness in improving financial performance. Therefore, the potential consequences of this issue are significant, as organizations may continue to invest resources in CSR activities without clear evidence of their impact on financial performance, potentially diverting resources from other areas that may have a more direct impact on financial results.

For all organizations, it is crucial to implement CSR programs that aim to deal with not only economic problems but also social and environmental problems as well as the well-being of society in general [23]. Our findings are consistent with previous research showing that social responsibility positively impacts customer satisfaction, image, reputation, and organizational performance [11,12,13,14,15,16,17,20,21,111,112,117]. Moreover, CSR actions, enhanced by adequate sustainability accounting and reporting, support organizations in managing sustainable development effectively [118].

This study takes a perceptual approach to examine the relationship between CSR and financial performance, looking at how employees, customers, investors, and creditors perceive the impact of CSR on critical variables such as reputation, employee attraction and retention, client attraction and loyalty, and access to capital. This study aims to provide a better understanding of the indirect effects of CSR on financial performance through these mediating variables and the direct impact of CSR on financial performance as perceived by stakeholders. By doing so, the study seeks to inform organizations about CSR’s potential benefits and drawbacks as a strategic tool for improving financial performance.

This paper has several contributions to the CSR literature. First, this study advocates the positive impact of CSR on financial performance. Organizations can obtain better financial performance through well-trained and loyal employees, loyal customers, and a positive market reputation. This finding aligns with economic theories in which it is posited that organizations that treat their employees and customers with respect and integrity are more likely to have better financial performance than those that do not. Second, the study emphasizes the importance of employee attraction and retention, customer attraction and loyalty, and reputation among investors and creditors for financial performance. Loyal employees lead to better productivity, loyal customers generate higher sales revenue, and confident lenders and investors ensure more accessible access to capital.

In conclusion, following an empirical study of a perceptual nature, this paper demonstrates that organizations’ social responsibility can significantly impact their financial performance both directly and mediated through the attraction and retention of employees, customer loyalty, and reputation. These theoretical implications are significant for company managers and researchers dealing with issues related to CSR.

### 5.2. Managerial Implications

CSR represents a strategic practice that can generate beneficial effects in relationships with stakeholders (attracting and retaining employees, attracting loyal customers, attracting and keeping investors, and increasing creditors’ trust). All of these effects are added to the direct effects of CSR on financial performance. This empirical study showed, through statistical results, that in employees’ perceptions, CSR, reputation, attraction and retention of employees, and attraction and loyalty of customers, more accessible access to capital can be significant predictors of organizational financial performance. In a world where information flows instantly and social networks contribute to its rapid spread, the reputation and image of organizations are essential for sustainable organizational performance.

The study results suggest that organizations that invest in CSR have better financial performance. CSR must be integrated into organizations’ strategies to achieve the most significant impact. Organizations should pay more attention to attracting and retaining employees through CSR practices.

Employees who perceive that their organization has greater social responsibility are likelier to stay with it and contribute to its financial performance. Employee involvement in CSR projects can also improve an organization’s financial performance by increasing productivity, employee morale, and engagement. Clients’ attraction and loyalty are essential factors in maintaining financial performance. CSR practices can improve customers’ perception of an organization and increase loyalty. Organizations should consider this and invest in CSR practices relevant to their customers. In addition, investors are interested in the CSR practices of the organizations in which they invest. Transparency in reporting CSR activities can improve an organization’s reputation and strengthen investor confidence. Therefore, organizations must report their CSR activities transparently and publicize relevant information to investors and other stakeholders.

Organizations with a good reputation for social responsibility attract more investors and can obtain financing at lower costs. Organizations that pay close attention to CSR practices can achieve significant intangible benefits in employee attraction and retention, client attraction and loyalty, investor attraction, and improved organizational reputation that translate into tangible benefits through increased financial performance.

### 5.3. Limitations and Further Research

Although all research hypotheses were validated, this empirical study presents several limitations that can be addressed and overcome in further investigations. First, although the sample is representative, it only targets a category of stakeholders (employees). In addition, the study was conducted on the employees of Romanian organizations, which can generate cultural particularities related to understanding the concept of social responsibility. The diversification of respondents from various countries and categories of stakeholders in future studies may increase the generalization of the research results. Employee perceptions may also vary based on their position and experience and may be influenced by external factors such as business cycles and legislative and fiscal changes. Future research may also examine differences in employee perceptions based on organizational size, industry sector, and level of management involvement in CSR. Second, other mediating variables, such as communities and public authorities, can be added in future research to establish a more inclusive framework on the influence of nontangible elements on performance, providing a new perspective on the benefits of CSR. Third, the research can have a longitudinal valence that would allow studying the effects of CSR on financial performance manifested over time.

## 6. Conclusions

In the current turbulent environment characterized by globalization and increased competitiveness, CSR has become a strategic practice differentiating organizations. Social responsibility programs affect employees and organizations, making employees’ perceptions vital for adequate implementation of CSR actions and having an essential role in organizations’ financial success. Therefore, employees must understand and identify with the CSR framework. This paper evaluates employees’ perceptions concerning social responsibility, financial performance, and a series of mediating variables that affect the relationship between the two constructs.

The paper suggests, starting from the empirical study findings, more significant involvement of employees in CSR actions, increasing employee awareness regarding the benefits that CSR brings to employees, customers, investors, creditors, and the community. The concerted CSR actions of all organizations improve employees’ quality of life, communities, and society. CSR must start with employees as internal stakeholders and extend into relationships with all stakeholders.

## Figures and Tables

**Table 1:** Frequencies of sociodemographic variables.

Variable	Answer Options	Frequency	Percent
Gender	Male	203	47.1
	Female	228	52.9
Age	18–30 years	46	10.7
	31–40 years	132	30.6
	41–50 years	126	29.2
	Over 50 years	127	29.5
Education	High school	47	10.9
	Bachelor	246	57.1
	Master or PhD	138	32.0

Source: Developed by the authors using SPSS v27.

**Table 2:** Questionnaire structure.

Latent Variables	Items	Scales
Demographic variables	Gender	Male (1), Female (2)
	Age	18–30 years (1), 31–40 years (2), 41–50 years (3), over 50 years (4)
	Education	High school (1), Bachelor (2), Master or PhD (3)
CSR	CSR level	Strongly agree (5) Partially agree (4) Moderate (3) Partially Disagree (2) Strongly disagree (1)
	CSR regularity	
Employees’ attraction and retention	Employees’ attraction	
	Employees’ retention	
Clients’ attraction and loyalty	Clients attraction	
	Clients loyalty	
Access to capital	Capital lenders	
	Capital investors	
Reputation	Reputation level	
Perceived performance	Net profit compared to last year	The substantial increase over the previous year (5) Increase compared to the previous year (4) Same as last year (3) The decrease compared to the previous year (2) The substantial decrease compared to last year (1)
	Turnover compared to last year	
Expected performance	Net profit compared to expectations	Much higher than expected (5) Greater than expectations (4) Up to expectations (3) Less than expected (2) Much lower than expected (1)
	Turnover compared to expectations	

Source: Developed by the authors using SPSS v27.

**Table 3:** Descriptive statistics.

Variable	N	Min	Max	Mean	Std. Deviation	Skewness	Kurtosis
Gender	431	1	2	1.53	0.500	−0.117	−1.996
Age	431	1	4	2.77	0.990	−0.202	−1.078
Education	431	1	3	2.21	0.621	−0.176	−0.561
CSR level	431	1	5	3.74	0.990	−0.388	−0.507
CSR frequency	431	2	5	4.02	0.936	−0.545	−0.722
REPUTATION	431	1	5	3.70	0.871	−0.043	−0.684
CLIENTS’ attraction	431	1	5	3.87	0.920	−0.485	−0.322
CLIENTS’ loyalty	431	1	5	3.75	0.978	−0.471	−0.365
CAPITAL lenders	431	2	5	3.96	0.870	−0.482	−0.488
CAPITAL investors	431	1	5	3.88	0.942	−0.528	−0.438
EMPLOYEES’ attraction	431	1	5	3.74	1.026	−0.539	−0.212
EMPLOYEES’ retention	431	2	5	3.84	0.991	−0.418	−0.884
Net profit compared to last year	431	1	5	3.56	0.960	−0.120	−0.792
Turnover compared to last year	431	1	5	3.82	0.870	−0.477	−0.151
Net profit compared to expectations	431	1	5	3.86	0.936	−0.365	−0.721
Turnover compared to expectations	431	1	5	3.35	1.276	−0.235	−1.023

Source: Developed by the authors using SPSS v27.

**Table 4:** Model reliability.

	Cronbach’s Alpha	Composite Reliability	Average Variance Extracted (AVE)
Access to capital	0.701	0.870	0.769
CSR	0.737	0.883	0.791
Clients’ attraction and loyalty	0.718	0.876	0.779
Employees’ attraction and retention	0.715	0.875	0.778
Expected performance	0.887	0.947	0.899
Perceived performance	0.796	0.907	0.830
Reputation	1.000	1.000	1.000

Source: Developed by the authors using SPSS v27.

**Table 5:** Discriminant validity.

	Access to Capital	CSR	Clients Attraction and Loyalty	Employees Attraction and Retention	Expected Performance	Perceived Performance	Reputation
Access to capital	0.877						
CSR	0.759	0.889					
Clients’ attraction and loyalty	0.563	0.613	0.882				
Employees’ attraction and retention	0.722	0.811	0.615	0.882			
Expected performance	0.788	0.840	0.668	0.840	0.948		
Perceived performance	0.756	0.826	0.667	0.831	0.858	0.911	
Reputation	0.597	0.560	0.550	0.556	0.669	0.634	1.000

Source: Developed by the authors using SPSS v27.

**Table 6:** Path coefficients.

	Coefficients Path (c)	Standard Deviation	t Statistics	*p*-Values
CSR -> Expected performance	0.286	0.039	7.405	0.000
CSR -> Perceived performance	0.292	0.045	6.482	0.000
CSR -> Clients’ attraction and loyalty	0.613	0.033	18.352	0.000
CSR -> Employees’ attraction and retention	0.811	0.016	51.331	0.000
CSR -> Access to capital	0.759	0.020	38.102	0.000
CSR -> Reputation	0.560	0.035	16.066	0.000
Clients’ attraction and loyalty -> Expected performance	0.102	0.030	3.433	0.001
Clients’ attraction and loyalty -> Perceived performance	0.129	0.035	3.638	0.000
Employees’ attraction and retention -> Expected performance	0.322	0.035	9.122	0.000
Employees’ attraction and retention -> Perceived performance	0.347	0.041	8.413	0.000
Access to capital -> Expected performance	0.183	0.032	5.691	0.000
Access to capital -> Perceived performance	0.137	0.039	3.530	0.000
Reputation -> Expected performance	0.165	0.027	6.078	0.000
Reputation -> Perceived performance	0.125	0.030	4.197	0.000

Source: Developed by the authors using SPSS v27.

**Table 7:** Specific indirect effects.

	Coefficients Path (c)	Standard Deviation	t Statistics	*p*-Values
CSR -> Employees’ attraction and retention -> Perceived performance	0.282	0.034	8.388	0.000
CSR -> Access to capital -> Expected performance	0.139	0.025	5.636	0.000
CSR -> Reputation -> Perceived performance	0.070	0.017	4.140	0.000
CSR -> Reputation -> Expected performance	0.092	0.016	5.666	0.000
CSR -> Clients’ attraction and loyalty -> Expected performance	0.062	0.019	3.282	0.001
CSR -> Access to capital -> Perceived performance	0.104	0.030	3.461	0.001
CSR -> Employees’ attraction and retention -> Expected performance	0.261	0.029	9.074	0.000
CSR -> Clients’ attraction and loyalty -> Perceived performance	0.079	0.023	3.460	0.001
CSR -> Employees’ attraction and retention -> Perceived performance	0.282	0.034	8.388	0.000
CSR -> Access to capital -> Expected performance	0.139	0.025	5.636	0.000
CSR -> Reputation -> Perceived performance	0.070	0.017	4.140	0.000
CSR -> Reputation -> Expected performance	0.092	0.016	5.666	0.000

Source: Developed by the authors using SPSS v27.

**Table 8:** Model predictability.

	RMSE	MAE	Q^2^
Access to capital	0.656	0.534	0.574
Clients’ attraction and loyalty	0.797	0.635	0.371
Employees’ attraction and retention	0.589	0.471	0.656
Expected performance	0.547	0.451	0.704
Perceived performance	0.567	0.463	0.681
Reputation	0.833	0.691	0.311

Source: Developed by the authors using SPSS v27.

## Data Availability

Not applicable.
